# Supplementary material for: A high-resolution mRNA expression time course of embryonic development in zebrafish
Source: eLife. 2017 Nov 16;6:e30860. doi: 10.7554/eLife.30860 (PMC5690287; doi:10.7554/eLife.30860)
Supplement: Supplementary file 6. [file elife-30860-supp6.zip › biolayout-clusters-files/Cluster182-genes.html]

Cluster182


# Cluster182: Genes

| Ensembl ID | Gene Name | Chr | Start | End | Biotype |
| --- | --- | --- | --- | --- | --- |
| ENSDARG00000058280 | ENSDARG00000058280 | 8 | 12998438 | 13026654 | protein\_coding |
| ENSDARG00000101743 | ENSDARG00000101743 | 5 | 56977002 | 56980612 | protein\_coding |
| ENSDARG00000020224 | ERGIC2 (1 of many) | 12 | 30245677 | 30251718 | protein\_coding |
| ENSDARG00000058992 | cers2b | 16 | 1338362 | 1373218 | protein\_coding |
| ENSDARG00000003411 | foxa2 | 17 | 42290592 | 42293092 | protein\_coding |
| ENSDARG00000059053 | slc13a4 | 4 | 4795123 | 4823579 | protein\_coding |
| ENSDARG00000101906 | zgc:113442 | 6 | 915534 | 923462 | protein\_coding |
| ENSDARG00000077248 | zgc:171459 | 25 | 19568670 | 19570228 | protein\_coding |
